# Defensive strategies of Norway spruce and Kurile larch heartwood elucidated on the micron-level

**DOI:** 10.1038/s41598-021-01590-y

**Published:** 2021-11-15

**Authors:** Sophie Füchtner, Sara Piqueras, Lisbeth Garbrecht Thygesen

**Affiliations:** 1 Institute for Geoscience and Natural Resource Management, University of Copenhagen, Rolighedsvej 23, 1955 Frederiksberg, Denmark; 2 MS-OMICS, Bygstubben 9, 2950 Vedbæk, Denmark

**Keywords:** Imaging, Optical spectroscopy, Plant sciences, Secondary metabolism

## Abstract

To decarbonize the building sector, the use of durable wood materials must be increased. Inspiration for environmentally benign wood protection systems is sought in durable tree species depositing phenolic extractives in their heartwood. Based on the hypothesis that the micro-distribution of extractives influences durability, we compared the natural impregnation patterns of non-durable, but readily available Norway spruce to more durable Kurile larch by mapping the distribution of heartwood extractives with Confocal Raman Imaging and multivariate data decomposition. Phenolics of both species were associated with hydrophobic oleoresin, likely facilitating diffusion through the tissue. They accumulated preferentially in lignin-rich sub-compartments of the cell wall. Yet, the distribution of extractives was found not to be the same. The middle lamellae contained flavonoids in larch and aromatic waxes in spruce, which was also found in rays and epithelial cells. Spruce-lignans were tentatively identified in all cell types, while larch-flavonoids were not present in resin channels, hinting at a different origin of synthesis. Larch-oleoresin without flavonoids was only found in lumina, indicating that the presence of phenolics in the mixture influences the final destination. Together our findings suggest, that spruce heartwood-defense focuses on water regulation, while the more efficient larch strategy is based on antioxidants.

## Introduction

The global production of building materials emits about 4–5 times as much CO_2_ as global air traffic. A green transition of the construction sector is therefore urgently needed^1^. One solution is to increase the use of wood, which is a renewable material with significantly lower climate impact than other building materials such as concrete and steel^2^. In Europe, Norway Spruce (*Picea abies*) represents an important resource for such initiatives due to its availability^3^. However, spruce wood is susceptible to degradation by fungi, while trees from the genus *Larix* generally produce more durable wood, even though the wood of these two genera is structurally similar. The explanation lies in the identity, amount and distribution of so-called extractives deposited within the heartwood of the living trees. They protect the main wood biopolymers against fungal decay, and appear to be among the compounds first targeted during initial fungal decay, as published recently in this journal^4^. Here we studied the differences in extractives between *Picea* and *Larix* with the long-term aim of developing cost-efficient methods based on bio-mimicking for protection of non-durable spruce wood against fungal decay.

Similar to harvested wood, the heartwood at the center of the trunk of living trees consists of dead tissue, which cannot react to wood-degrading organisms^5^. This is opposed to sapwood, where the living tissue creates reaction zones to limit the spread of a given pathogen^5^. To protect the heartwood, parenchyma cells form species-specific extractives that diffuse into the surrounding tissue (tracheids) while heartwood is formed^6^. Phenolic extractives are considered to be the main contributors to resistance against degradation by fungi and bacteria via their antioxidative, metal chelating, radical scavenging and/or biocidal properties^6–9^. Lignans and flavonoids are the phenolics produced by spruce and larch, respectively. In addition, oleoresin is produced in the sapwood by ray parenchyma cells and epithelial cells surrounding resin channels. Oleoresin is a sticky, hydrophobic mixture of a variety of mono- and sesquiterpenes, i.e. resin acids (RAs), fatty acids (FAs) and their esters. Its composition may change in response to a fungal attack, as the constituents also display antioxidative properties^8,10^. When heartwood is formed, oleoresin diffuses into the surrounding tracheids^8,11^. *Larix* additionally deposits large amounts of arabinogalactan (ArGal) in cell lumina upon heartwood formation, but its purpose is not clear^12,13^.

Extractives have been studied intensively since the 1960ies and their heterogeneous distribution within the wood tissue has been recognized^8,10^. They have been found in resin channels and rays, in the lumen of tracheids, lining the cell wall (CW) and filling pits (cell connections) ^7,8,10,14^. Increasing evidence shows that extractives may also be located inside the cellulose-rich CW and lignin-rich compound middle lamellae (CML, middle lamellae + primary wall), i.e. in Larix^15,16^, Pinus^11,17^ and others^18,19^. Observations that extractives have a greater effect on wood properties when located inside the CW, rather than in the lumen of cells^8^, have led to the idea that the micro- or nano-distribution of extractives is important for their defensive functions in heartwood^7,8,10^.

Confocal Raman spectroscopy is a method that has gained importance in the chemical mapping of biological tissue. It combines vibrational spectroscopy with a confocal microscope, allowing the acquisition of spectra with a spatial resolution in the sub-micrometer range. Decomposition of the data into putative pure component spectra and their concentrations at each image pixel can then be achieved using Multivariate Curve Resolution—Alternating Least Squares (MCR-ALS)^20,21^.

**Figure 1:**
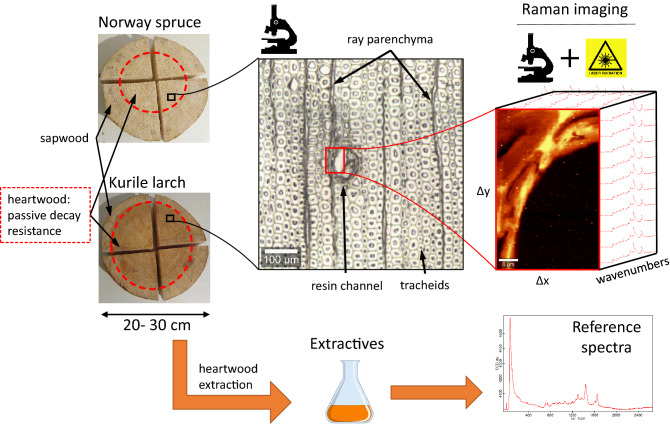
Outline of the study. Norway spruce (*Picea abies*) and Kurile larch (*Larix gmelinii var. japonica*) are conifers that produce wood with different levels of natural resistance against fungal degradation. The secondary metabolites that cause the resistance are termed extractives and can be extracted with solvents of different polarity. Phenolic extractives are produced upon heartwood formation, causing a dark coloration of the heartwood tissue in more resistant species (Kurile larch). Among other factors, the micro-distribution of extractives within the tissue is thought to contribute to the performance of the wood against fungal attack. Imaging with confocal Raman spectroscopy allows the detection of extractives in native tissue on a nanometer scale. Identification is aided by comparison of resolved image spectra (MCR-ALS) to reference spectra obtained from extracts.

In this study (Fig. 1), we used this approach to compare the native micro-distribution of heartwood extractives in a moderately durable conifer species (Kurile larch) with those found in a non-durable one (Norway spruce) with similar anatomy. Knowledge about the local extractive composition in the tissue of different conifers could aid the advancement of artificial wood protections systems, by revealing natural protection strategies used by different species. In this context, our aim was threefold: Firstly, we would like to clarify whether there are differences in the micro-distribution of extractives between the two conifers. Secondly, whether the composition and chemical structure of extractives could cause the observed micro-distribution. And thirdly, what defensive mechanisms could tentatively be inferred from these observations.

## Results

### Cell wall components and background signals

Three to five Raman images for each region (tracheids, rays, resin channels) were obtained from two spruce trees and one larch tree and combined into group-wise multisets for MCR-ALS analysis (schematically shown in the supplementary information (SI), Fig. S1). All spruce data were obtained at 532 nm excitation, while larch data were obtained at 785 nm to avoid fluorescence issues.

The commonly known CW polymer spectra of lignin, and cellulose in two orientations (perpendicular (⊥) and parallel (‖) crystal orientation relative to the incident beam polarization) were identified in all datasets. Additional background signals were due to water from sample preparation (spruce) and CaF_2_ from the cover-glass and noise/water (larch).

The bands used for identification are summarized in Table 1 and the respective component spectra are shown in the SI (Fig. S3). The distribution of CW polymers followed the trends previously shown for Raman images of tracheids in spruce^22^, pine^17,23^, beech^24^ and poplar^25^ and can be viewed in Fig. 4 (C1–C4) and Fig. 5 (C1–C3 and C7–C8). Please refer to the SI (Sect. 4) for a more detailed description.

**Figure 2:**
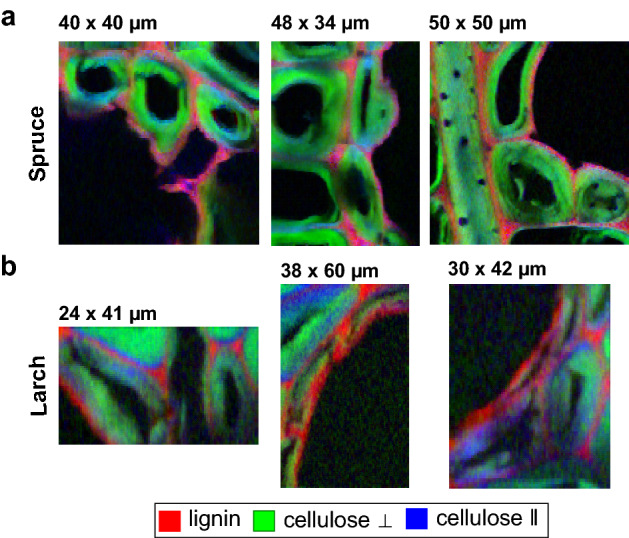
Overlay of the cell wall polymer distribution of epithelial cells obtained via MCR-ALS analysis of the Raman images of (**a**) Norway Spruce and (**b**) Kurile larch. Although lignin and cellulose with two crystal orientations (perpendicular (⟂) and parallel (∥) to incident beam polarization) are present in the cell walls, the more intense blue coloration of larch epithelial cells indicated a higher contribution of this component compared to spruce. The intensities of each component were set to the range 0–1.

We found structural differences between species in the relative CW composition of epithelial cells surrounding the resin channels in the two conifers (Fig. 2). In spruce, the parallel oriented cellulose component was spectrally not completely resolved (Fig. S3), a sign that it contributed little to the overall image. This is also reflected in the more intense coloration of the parallel oriented cellulose component in larch epithelial cells, in comparison to spruce. Furthermore, we found that lignin surrounds epithelial cells, also on the side facing the intercellular space making up the channel void.

### Extractive identification and localization

**Figure 3:**
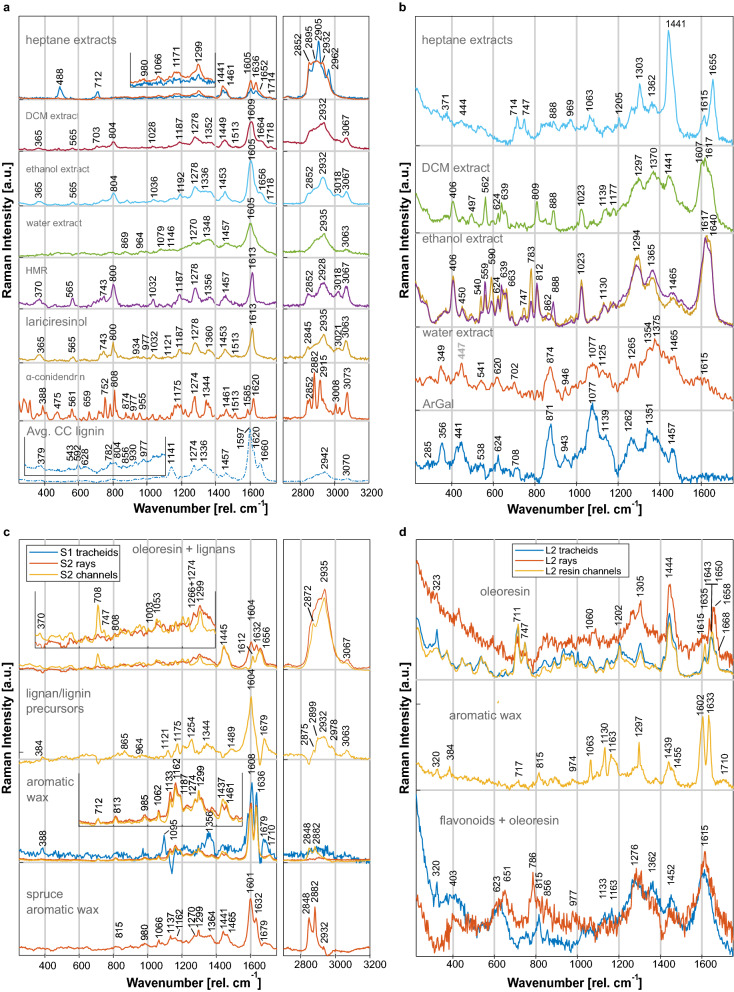
Extractive references and MCR-ALS extractive components of Norway spruce and Kurile larch. Spectra have been normalized for comparison and where relevant, spectra have been zoomed in. (**a**) Spruce references (532 nm excitation): overlay of spectra of heptane extracts of spruce 1 + 2 (blue) and spruce 2 (red); dichloromethane (DCM) extract of spruce 2 after prior heptane extraction; ethanol extract of spruce 2 after heptane and DCM extraction; water extract of spruce 2 after all previous solvents; isolated lignans: hydroxymatairesinol (HMR), lariciresinol and α-conidendrin; average spectrum of spruce cell corner (CC) lignin with zoom on the 250–1100 cm^-1^ region. (**b**) Larch references (785 nm excitation): heptane extracts, DCM extract, ethanol extract, water extract and pure arabinogalactan (ArGal). (**c**) Spruce MCR-ALS extractive components: Oleoresin with traces of lignans, lignans in the compound middle lamellae (CML), aromatic wax and spruce aromatic wax; (**d**) Larch MCR-ALS extractive component: oleoresin, aromatic wax and flavonoids with traces of oleoresin.

To enable interpretation of the components found by MCR-ALS, reference spectra of various extracts and pure chemicals were obtained and are shown in Fig. 3a, b for spruce and larch, respectively. A detailed analysis of the reference spectra can be found in the SI (Sect. 5). Resulting from this analysis, the most important bands for identification of individual chemical groups are summarized in Table 1 for each of the laser wavelengths used.

**Figure 4:**
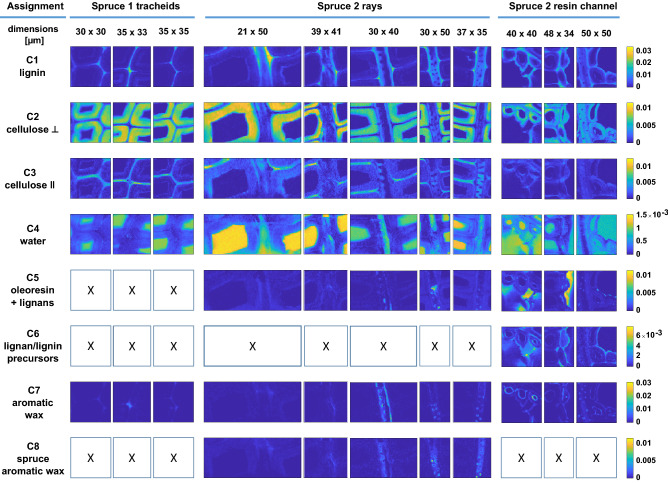
Distribution maps of components obtained from group-wise MCR-ALS analysis of Raman images of Norway spruce tracheids, rays and resin channels. White squares with an “x” are components that were not found in the respective image. The images sizes are not to scale, but each row shares the same intensity scale.

The MCR-ALS component spectra identified as extractives (in total 4 for spruce and 3 for larch) are shown in Fig. 3c, d. Their distribution can be viewed in Figs. 4 (C5–C8) and 5 (C4–C6) and in SI Sect. 6 as an overlay.

**Figure 5:**
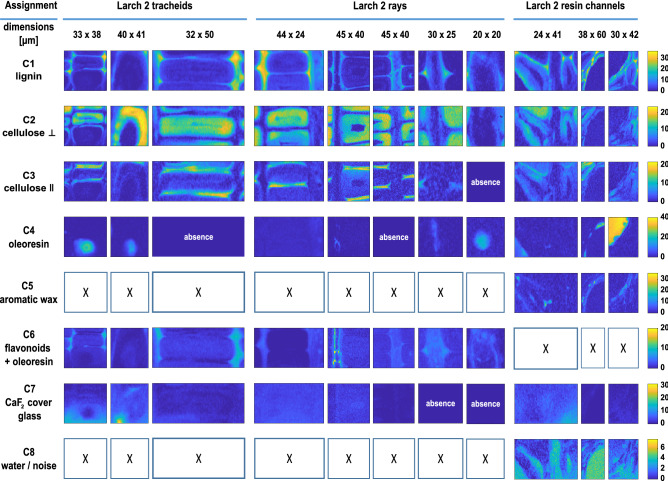
Distribution maps of components obtained from group-wise MCR-ALS analysis of Raman images of Kurile larch tracheids, rays and resin channels. White squares with an “x” are components that were not found in the respective image. Fields marked with “absent” are components that were not present only in some of the images of a given multiset. The images sizes are not to scale, but each row shares the same intensity scale.

**Figure 6:**
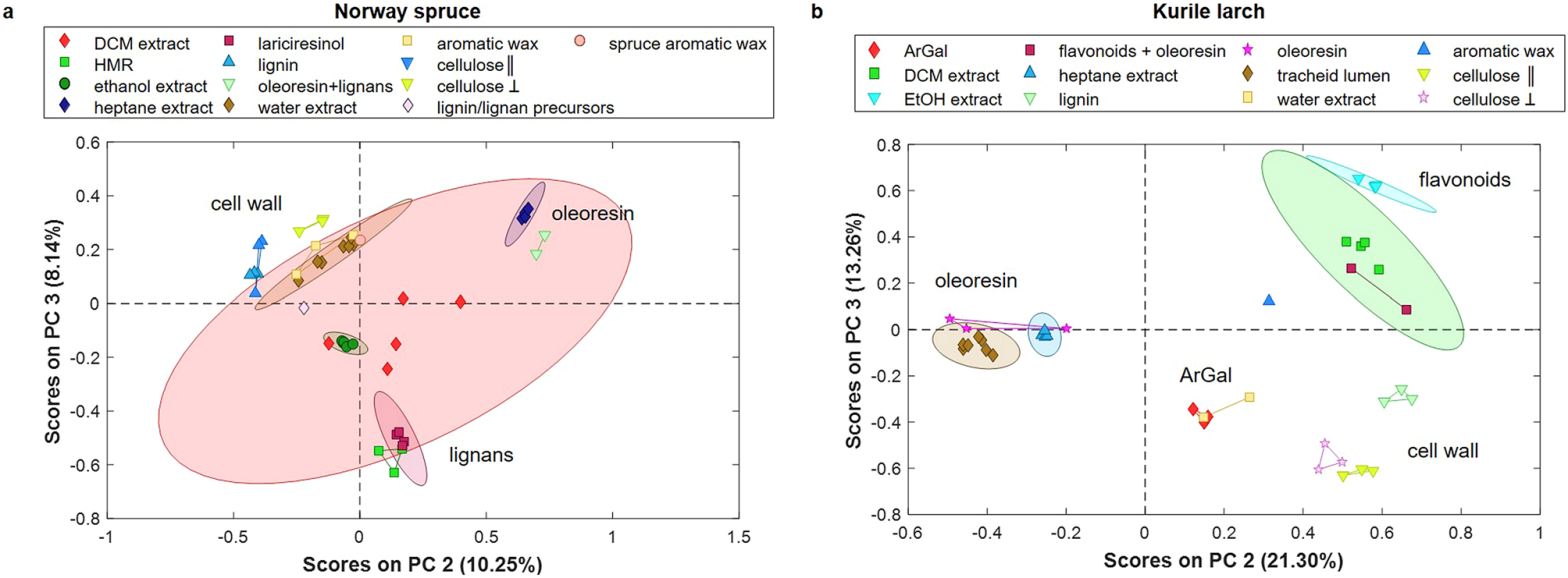
Sample scores of a principal component analysis performed on the MCR-ALS components and references of (**a**) Norway spruce and (**b**) Kurile larch. Principal components (PCs) 2 and 3 are shown; the rest can be viewed in the supplementary information Sect. 6.

Oleoresin was identified as one of two extractive components common to spruce and larch, showing great similarity to the heptane extracts of the respective species (Fig. 3). The larch oleoresin components corresponded perfectly with the extracts, containing mostly isopimaric-type resin acids, FAs and their glycerol esters. This was reflected in the scores of a principal component analysis (PCA) performed on references and extracts (Fig. 6b) where the heptane extracts were very close to the oleoresin component found by MCR-ALS. Furthermore, several single spectra of deposits in various tracheid lumina also associated to the latter, proving that several tracheids are filled with pure oleoresin in larch.

Some differences were observed in spruce oleoresin. The component obtained for spruce channels shows several bands characteristic for dehydroabietic acid, FAs and their glycerol esters, while the same component in the rays was more similar to the extracts, potentially showing more oxidized forms of RAs (Table 1, SI Sect. 5). In both components, the profile of the aliphatic C-H stretches changed, as the maximum was now observed at 2935 cm^-1^, rather than at 2895 cm^-1^ as in the heptane extract. This may be the result of a different lipid composition, i.e. higher abundance of triacylglycerols^27^.

**Table 1:** Raman marker bands of the cell wall polymers present in wood, as well as of several substances found in extracts and MCR-ALS components of Norway spruce and Kurile larch.

Substance	Marker bands as observed in this study (cm^−1^)	References
Lignin	3070 (w), 2945 (w), 1660 (m), 1620 (sh), 1597 (vs), 1457 (w), 1274 (m), 1141 (m)	^24,26^
	1660 (m), 1597 (s), 1457 (w), 1274 (m), 1141 (m)	
Cellulose ⊥	2895 (s), 1372 (m), 1336 (m), 1120/1124 (m), 1095 (w), 384 (m)	^24^
	1372 (m), 1336 (m), 1120/1124 (m), 1095 (m), 384 (s)	
Cellulose ‖	2902 (m), 1095 (m), 384 (w)	^22,25^
	1096 (s), 378 (w)	
Lipids	**fatty acids:** 2852–2962 (vs), 1652–1656 (m, unsaturated), 1461 (m), 1441/1445 (m), 1303 (w); **triacylglycerols:** 2935 (vs), 1063–1066 (w); **crystalline waxes:** 2905 (s), 1297 (w, sharp), 1121 (w, sharp), 1063 (w, sharp), 980–985 (w)	^27–29^
	**fatty acids:** 1658 (m), 1441/1444 (s), 1305 (m); **triacylglycerols:** 1063 (w); **crystaline waxes:** 1297 (m, sharp), 1130 (m, sharp), 1063 (m, sharp), 974 (w, unsaturated)	
Resin acids	**general skeleton and abietic-type:** 1650 (m), 747 (w), 712 (m); **dehydroabietic acid:** 1612 (w), 708 (m), 747 (w), 708 (m); **isopimaric acid:** 1632–1636 (m), 1433 (m), 712 (w); **oxidized resin acids:** 1604 (s), 1636(s), 1710 (w)	^17,30^
	**general skeleton and abietic-type:** 1650 (m), 747 (w), 714 (w); **dehydroabietic acid:** 1615 (w), 747 (w), 711 (m); **isopimaric acid:** 1668 (sh), 1635–1643 (m), 1439 (m), 714 (m); **oxidized resin acids:** 1602 (s), 1636 (s), 1710 (w)	
Para-hydroxy cinammic acid and derivatives	**coumaric acid:** 1636 (s), 1605 (s), 1254 (m), 1204 (m), 1171 (m), 865 (w); **ferulic acid:** 3069 (w), 1628 (m), 1608 (s), 1274 (w), 813 (w); 711 (w); **ferulates:** 3062–3068 (w, alkyl ester), 1670–1690 (w, steryl ester), 1676–1684 (w, alkyl ester), 1632–1634 (s), 1133 + 1162 + 1187 (w, propyl ester), 1122–1127 (w, steryl ester), 1118–1134 (w, alkyl ester)	^28,31–34^
	**ferulic acid:** 1602 (s), 815 (w)**; ferulates**: 1633 (s), 1439 (m), 1163 (m), 1130 (m)	
Lignans	3067 (m), 3018–3021 (w), 2932–2935 (m), 1613/1620 (s, isolated lignan), 1605 (s, lignan in extract), 1453–1461 (w), 1350–1360 (w), 1274–1278 (w), 1187 (w), 1032 (w), 800 (m), 565 (w), 365 (w)	^33^
Flavonoids	1640 (s), 1617 (s), 1607sh, 1365 (m), 1294 (m), 1023 (M), 812 (w), 783 (m), 690–540 (w-m), 406 (m)	^35–37^
Arabinogalactan	1457(m), 1351 (m), 1262 (m), 1077 (s), 871 (s), 441 (m), 356 (m)	^38^
CaF_2_	323 (s, sharp)	^39^

Cellulose orientation as indicated in the table is relative to the incident beam polarization. Rows with a grey background show the bands found in Kurile larch using an excitation wavelength of 785 nm. Rows in white show bands found in Norway spruce at excitation wavelength 532 nm. The intensities have been assigned in approximate relation to the highest peak in the respective spectrum.

In spruce, an important difference to the heptane extracts was the presence of an aromatic C-H stretch at 3067 cm^-1^ in the oleoresin component (Fig. 3c), which was also observed in an average spectrum of the resin in channels (SI Fig. S2). The dichloromethane (DCM) and ethanol extracts, as well as isolated lignans i.e. hydroxymatairesinol (HMR), lariciresinol (Fig. 3a) and secoisolariciresinol^33^ presented this band too. The band shift to 2935 cm^−1^ could also be caused by the presence of HMR. The C-H stretching region of RAs has not been published to our knowledge, but personal communication with Victoria Beltran and Peter Bock revealed that aromatic and conjugated RAs display this band at somewhat lower frequencies, making it less likely that this band originates from RAs. Aromatic C=C stretches were evident in the ray signal at 1604 cm^-1^, which is lower than for the isolated lignans or RAs^17,30^, but corresponds well with the extracts. Furthermore, the ray component shows a broad combination band from 1310 to 1380 cm^−1^, also seen in the spruce ethanol extract. A counter-indication for the presence of lignans is the lack of the medium-intense band at 800 cm^−1^. The scores of the PCA performed on spruce data (Fig. 6a) show the similarity of the extract to the native component, but the oleoresin/lignan component was slightly shifted towards the isolated lignans. We thus tentatively identify this component to be a mixture of oleoresin with small amounts of lignans.

A potential consequence of this difference was the micro-distribution of this component, which was confined to lumen spaces in larch (i.e. in resin channels, ray parenchyma, pits and tracheid lumen, Fig. 5, C4), while it additionally spread throughout the CW layers of spruce tracheids, with higher concentrations in the S1 and S3 layers (Fig. 4, C5).

Another indication for the presence of lignans in the oleoresin of spruce heartwood was seen in the resin channel images (Fig. 4, C6). Although spectrally not perfectly resolved, a distinct component was found in the middle lamellae of the epithelial cells in addition to the lignin component described above.

The strong band at 1604 cm^−1^ is a general indication of aromatic compounds in conjugation with C=C and/or C=O bonds^28^. Bands at 1254 (in phase C–X ring stretch), 1175 (aromatic C–H bend) and 865 cm^−1^ (ring breathing) indicate para-substituted coumaryl rings^40^. The band at 1679 cm^−1^ hints at the presence of alkyl or steryl esters of ferulic acid^31,34^, which could be similar for coumarates. Additionally, p-coumaric acid also has a band at 3067 cm^−1^^33^. Due to the lack of an intense doublet at 1601 and 1636 cm^−1^, ferulates are unlikely, but for coumaric acid and derivatives the band around 1636 cm^−1^ is less intense^28^ and may be hidden under the broad base of the 1604 cm^−1^ band. Para-coumaric acids, are known precursor molecules in the lignan/lignin biosynthetic pathways^41^. The CH stretching bands at 2899, 2932 cm^−1^ are not seen in p-courmaric acid^33^, but correspond well with lignans bands like HMR (2932 cm^−1^) and secoisolariciresinol (2899 cm^−1^, ^33^), which also have the aromatic C–H stretch at 3061–3063 cm^−1^. Furthermore, the aromatic C=C stretch of lignans was also broad and shifted to 1605 cm^−1^ in the extracts. In the PCA scores (Fig. 6a), this component is found in between the lignin component and ethanol extracts. Thus, the component is interpreted as a mixture of lignan/lignin precursors (i.e. coumaric acid and derivatives) and low amounts of lignans.

The second common extractive component of spruce and larch was likely an aromatic wax layer (Fig. 3c, d), very similar to the epicuticular layer also found covering the needles of Norway spruce trees^28^. In both species, the signal was found lining the lumen of epithelial cells (Figs. 4, C7 and 5, C5), but also appearing as droplets in larch epithelial cells. In spruce this component was additionally found lining the ray CW and in the torus of pits.

The most striking feature of this component was the aromatic doublet at 1608/1602 and 1636/1633 cm^-1^, which together with the bands at 712/717 cm^-1^ could indicate the presence of RAs and oxidation products thereof^30^. Ferulic acid may be identified based on the above-mentioned doublet and additional bands at 1162 (aromatic ring modes + O-CH_3_ stretch) and 813/815 cm^-1^ (ring modes) (Table 1). Alkyl or steryl ferulates may be evident from bands at 1679 and 1710 cm^-1^ (C = O stretches). In the spruce components, the presence of long chain hydrocarbons is evident from C-H_2_ stretching bands at 2848 and 2882 cm^−1^, which in case of the ray component have probably been fitted entirely to a similar component also found in the images (see below). The rest of the component is almost identical between the two species, showing lipidic substances (i.e. 1437–1461 cm^−1^). The relatively sharp appearance of the bands at 1299, 1130 and 1063 cm^−1^ are indicative of an ordered, crystalline configuration, as seen in waxes^29,32^.

Furthermore, the waxy component was found in the CML and CCs of the spruce tracheid images. Although cellulose concentrations are low in the CML/CC, the component showed some features of cellulose, which was also observed in the cuticular layer of spruce needles^28^.

A spectrally very similar component was also found as droplets in the rays and within pits. The lower contribution of the 1632 cm^−1^ band may indicate a different composition, i.e. by the presence of less saturated lipids, which could lead to a more fluid consistency of the substance. Also the concentration of phenolics may influence the latter^32^. This in turn could lead to a markedly different distribution as seen in Fig. 4 (C8). Note that the band at 1299 cm^−1^ is clearly sharp, still indicating an ordered state of the component. To differentiate between the two aromatic wax components, the former one will be referred to as aromatic wax and the latter as spruce aromatic wax.

In larch, another phenolic component was distributed intensely in the CML of tracheids and the ray lumen and somewhat lower in the tracheid CW layers (Fig. 5, C6). Contrary to spruce, it was not present in the resin channel images. The spectrum (Fig. 3d) indicated the presence of flavonoids via the broad aromatic ring stretch at 1615 cm^−1^ and the skeletal vibrations at 815 and 786 cm^−1^ also seen in the references (Fig. 3b). Furthermore, the C-H bending vibrations appeared at 1452 cm^−1^, which is shifted higher than observed for the hydrophobic extracts (1441 cm^−1^) and lower than observed in the flavonoid-rich ethanol extract. Thus, it is likely that this component showed the presence of lipids as well as flavonoids. The PCA performed on the larch components and references (Fig. 6b and SI Sect. 7) showed a close association of the DCM extract with this flavonoid component, confirming a mixture of oleoresin and flavonoids.

Due to the lack of the intense signals of arabinogalactan at 1077 and 871 cm^−1^ in larch images, it is unlikely that the polysaccharide was present within the CW or lumen spaces of our specimen. It probably washed out of the sample sections during sample preparation, due to its high water solubility. Nevertheless, the majority of other extractives was likely still present in the sample, as flavonoids are more hydrophobic than ArGal. For this reason, the present study cannot give insight on the composition of ArGal in the lumen spaces of larch and potential functionality arising from it.

## Discussion

Confocal Raman imaging, together with multivariate analysis tools, proved a powerful combination of techniques to identify native extractives and localize them within the heartwood tissue of Norway spruce and Kurile larch. Our findings were strengthened by comparison to reference spectra, but limited by the sample preparation in water, which lead to the loss of ArGal in larch samples.

### Extractive distribution is influenced by cell wall and extractive composition

The lower amounts of parallel oriented cellulose micro-fibrils in the epithelial cells of spruce compared to larch may have implications on the metabolites that can be secreted at the sapwood-stage, i.e. lignans were (tentatively) identified in and around them in spruce, but no flavonoids were found in larch. Whether this difference is a consequence of the cell wall thickening during heartwood formation^42^, or a property already in place in the sapwood needs to be clarified in the future.

The only phenolics-free extractive (larch oleoresin) was found exclusively in lumina, while all other extractives were found both within and outside the cell wall. This indicates that the type of molecules present in a mixture of extractives may influence the micro-distribution in wood. We showed a close association between oleoresin (hydrophobic mixture of FAs, RAs and sterols^43^) and the heartwood phenolics of both species, analogous to the observations in Scots pine^17,23^, which suggests a solvating role.

In our larch specimen, the ratio of oleoresin to flavonoids was about 4:1^43^, potentially explaining why the intense flavonoid component only showed minor signs of fatty acids. They were probably sourced from the rays, where the largest aggregates were observed, and which has also been seen in Japanese larch^15^. Flavonoids were also found throughout the tracheid tissue, with decreasing signal intensity from CML, followed by S1/ S3 and S2 layers. With this, they follow roughly the distribution of lignin within the tissue^10^. The flavonoid distribution agreed with earlier findings, where flavonoids were detected throughout the CW layers, most intensely in rays and CML, and in close association with lignin^16^.

In larch, a clear spatial overlap of oleoresin with flavonoids was only seen in the ray parenchyma cells. This suggests that these cells may be responsible for the production of flavonoids in larch, rather than epithelial cells. Accordingly, we hypothesize that oleoresin mixes with flavonoids within the ray parenchyma cells and that the oleoresin then facilitates flavonoid transport into the CWs. In support of this, it has been shown that the ray parenchyma cells contain precursor molecules of flavonoids in the sapwood, which gradually transform and spread into the tissue during heartwood formation^15,16^.

The lignan containing oleoresin component of spruce heartwood was dominated by oleoresin bands, although lignans should be present at almost equal concentration^43^. This may suggests that not all lignans are found within the oleoresin. This component was present as deposits in the resin channels, rays and pits, but was also seen as infiltration in the S3 layer of tracheid CWs, and weaker in the S2 layer. We also saw that the oleoresin found in the rays might be more oxidized or older than the one in the resin channel. Since the resin channel network is under pressure^5^, a potential reason could be that it is not interrupted due to heartwood formation, and thus fresh resin may reach the heartwood, in spite of the dead epithelial cells.

Coumaric acid derivatives and lignans were tentatively identified at the inner and outer surface of epithelial cells. One interpretation is that this component is the result of lignan synthesis and secretion and/or remnants of lignin synthesis that occurs during cell wall thickening of epithelial cells upon heartwood formation^42^. On the other hand, the joint appearance of these molecules coincides with earlier research in that lignan-coumarates were found in Norway spruce stemwood extracts^44^, although our data do not allow conclusions on covalent linkages between them. Lignan-coumarates are also abundant in the highly antioxidant callus resin of Norway spruce^44^, and thus this component may contribute to a chemical defense mechanism specific for resin channels.

A waxy layer, infused with RAs and ferulic-acid-type molecules, had the same distribution in epithelial cells of both species. A similar pattern was seen for RAs and tannins in Douglas-fir^19^. In spruce, the waxy layer was additionally found to cover ray cell walls, surrounding and filling pits, as well as occupying the CML between tracheids. As opposed to findings in Scots pine and Kurile larch, only precursor molecules of lignans were found in the CML of Norway spruce, while in the former cases, the end-products of their biosynthetic pathways (stilbenes and flavonoids, resp.) were found. Lipids were also co-localized with phenolics in the CML of pine^23^ and the PCA of our Kurile larch data suggested that the flavonoid component isolated from the images correlated very well with the DCM extract, which was previously shown to contain both, hydrophobic components (lipids) and flavonoids^43^. In support of this, earlier research has identified alkyl ferulates in Norway spruce^45^ and in Western larch^46^. Alkyl ferulates were also detected in the heptane extracts of our spruce specimens and in lower amounts in Kurile larch with GC–MS (data not published). The fact that this component was only detected in the resin channels of larch could explain why lower amounts were detected by GC. Alternatively, if present, the component could have been covered by large amounts of flavonoids in rays and CML of tracheids.

### Extractive location and the link to their function

According to Segmehl et al. (2018), the porous wooden CW allows for two routes of transportation of extractives: via pits and the cell lumen to the lignin-rich S3 layer, and through the lignin-rich intra-cellular space of the CML and CCs^47^, possibly reaching this area via blind pits that end at the intra-cellular space^48^. This route may be kinetically favored, explaining the high accumulation of extractives in the CML. Our data agree with previous observations that demonstrated higher extractive concentrations in the lignin-rich CW segments^16–18,23^. The lowest concentration of extractives was usually found in the S2 layer. A potential explanation could be the qualities of the micro-environment of the respective CW compartments, i.e. it seems reasonable that cell compartments rich in lignin are more hydrophobic than the cellulose-rich S2 layer, resulting in a higher affinity of phenolics towards this area^47^. Further, association with hydrophobic oleoresin may facilitate the diffusion of phenolics through this network^23^. Note that native resin has a relatively low viscosity due to the presence of native solvating molecules in the form of monoterpenes^8,10^, and usually in the form of solvents like ethanol in impregnation experiments^23,47^.

From a defense-strategic point of view, investing in the blockage of rays and pits with extractives seems advantageous, since hyphae are thought to initially move through rays and pits into tracheid lumina. As the next line of defense, protection of cellulose by surrounding it with extractive-infused lignin could be key to curb a potential fungal invasion, thus linking extractive location to their function. Protection of the lumen-sided S3 layer would hinder cellulose-feeding brown-rot fungi from reaching the S2 layer. Furthermore, protection of the CML seems important, as it is the most important structural unit for compression strength and stiffness of the stem^5^. Thus, preventing non-enzymatic oxidative degradation by brown-rot fungi and the enzymatic machinery of lignin-feeding white-rot fungi with large quantities of antioxidants in this area would continuously slow down the degradation process.

Several mechanisms for water barriers are set in place by the extractives, which is one of the primary factors to avoid fungal decay^43^. Especially the waxy component detected in all spruce images and epithelial cells of larch provides such functionality. In spruce needles^28^ and *Arabidopsis thaliana*^32^, a cuticular layer of very similar composition was found. It functions as a water barrier, and the p-hydroxy cinammic acids therein also present antioxidant properties^28,31,32^. Oleoresin and the spruce aromatic wax may assist the sealing of pits, further reducing the possibilities of water flow^23^. Extractives in and on the S3 layer likely contribute to a decreased wettability of the CW^14,17,45^. Albeit present at low concentrations, the phenolic extractives in the S2 layer are thought to contribute to the desiccation of the heartwood tissue by displacing the CW water^14,43^. Since a large part of various extracts were found to be polymeric^43,49,50^, it is likely that they polymerize in-situ, and perhaps even crosslink to the CW polymers^6,16,17^.

The role of ArGal remains uncertain. Although we were not able to show it, it is known, that ArGal is present in large quantities in the lumina of cells and may therefore act as a pathway blocker for microorganisms. On the other hand, since it is a polysaccharide, various microorganism may be able to draw energy from its consumption^13^.

Based on these results, we speculate that the primary heartwood defense strategy of Norway spruce is moisture regulation in all parts of the tissue, assisted by a variety of antioxidant phenolics. The more efficient strategy of Kurile larch appears to be blockage of rays and the infiltration of the CW layers with large quantities of antioxidant flavonoids, mixed with hydrophobic compounds that facilitate their diffusion into the tissue. The species’ strategies seem to converge on the preferential protection of lignin-rich CW layers—CML, S1 and S3.

## Conclusion

The combination of Confocal Raman Imaging and MCR-ALS decomposition of the data provided detailed insight into the sub-cellular distribution of heartwood extractives in Norway spruce and Kurile larch samples.

Among the resin secreting cells, lignans were tentatively identified in the CWs of epithelial cells and mixed with the oleoresin of resin channels and rays in spruce. In larch, flavonoids were only found in rays. This may hint at different origins of heartwood-phenolics synthesis. Epithelial cells of both conifers were covered by an aromatic wax component. The same substance was also found on and within the torus of pits in spruce, probably regulating water-content.

While the CCs and CML of larch were covered with large quantities of flavonoids in larch, an aromatic wax component, but not lignans, was found there in spruce. Flavonoids, lignans and potential precursors thereof were detected in the remaining cell wall, with higher intensity in the S1 and S3 layers. Thus, Norway spruce heartwood defense seems to focus on physical water regulation, while in Kurile larch, a chemical defense mechanism via large quantities of antioxidant flavonoids prevails.

The different distribution of larch oleoresin with and without flavonoids is a clear sign that the type of compounds present in a mixture influence the distribution within the wooden tissue. This was less apparent in spruce, as all detected components contained some form of phenolic compounds and were found within and/or outside the CW. Our data agree with previous studies in that, within the tracheid CWs of both conifers, heartwood-phenolics are associated with hydrophobic substances, and deposited preferentially (but not exclusively) in the lignin-rich CW layers (CCs, CML and S1/S3 layer).

## Methods

### Sampling and sample preparation

The wood specimens came from two Norway spruce (*Picea abies*) trees (named Spruce 1 and Spruce 2, 48 years old), and a Kurile larch (*Larix gmellinii var. japonica)* tree (65 years old). The trees were harvested in the Hørsholm forest in Denmark in 2017 with permission of the local municipality. The mature heartwood at 1.3 m stem height was isolated and cut to obtain sticks with the dimensions of 3–4 × 4–3 × 50 mm corresponding to the longitudinal, radial, and tangential directions. Then the material was freeze-dried. The detailed procedure can be found in Füchtner et al.^43^.

Using a microtome (Leica RM2255), several 10–15 µm thin cross-sections were prepared for Raman microscopy as described in Gierlinger et al.^51^. Due to problems with auto-fluorescence during measurements in the visible range, sections of larch were prepared for measurements in the near-IR region, by mounting them on CaF_2_ slides and coverslips, reducing signal interference during Raman measurements.

To complement the analysis with reference spectra, extracts of the milled heartwood of all four trees were prepared. Extraction was done sequentially with 99% heptane (physical, health and environmental hazard), dichloromethane (health hazard), 96% ethanol (physical and health hazard) and water using an Accelerated Solvent Extractor (Dionex ASE 350, Thermo Electron A/S, Scientific Instrument Division, 2650 Hvidovre, Denmark), as described in Füchtner et al.^43^. The liquid extracts obtained from spruce and larch were concentrated on a glass slide for Raman measurements. Furthermore, Patrik C. Eklund (Åbo Akademy University, Turku, Finland) kindly provided the reference lignans hydroxymatairesinol (HMR, racemic mixture), lariciresinol:cyclolariciresinol (9:1) and α-conidendrin isolated from Spruce knotwood. A spruce lignin reference was obtained as an average spectrum (n = 6) from the cell corner of one of the images. Finally, a reference spectrum was also obtained for ( +)-arabinogalactan from larch wood (Sigma-Aldrich). All solid reference samples were put on a glass slide without any further fixation.

### Raman measurements

Raman measurements were conducted using a WITec alpha 300R confocal Raman microscope (WITec GmbH, Lise-Meitner-Str. 6, D-89081 Ulm, Germany). Images of rays with surrounding tracheids, parts of resin channels and tracheids next to rays were taken. For spruce, tracheid images were taken from tree 1 and the other images from tree 2. Table S1 in the SI shows an overview of the recorded images.

Norway spruce wood samples and reference substances were analyzed with the 532 nm excitation laser (diode, green) at 10 mW laser power. The spectrometer for this laser (UHTS300 spectrometer VIS) was equipped with a back-illuminated CCD camera with Peltier cooling to − 60 °C and a 600 g/mm grating, resulting in a spectral resolution of 3.8 cm^−1^. The images of the wood samples were taken using a 100 × oil immersion objective (Zeiss “N-Achroplan”, NA = 1.2, WD = 0.29 mm; Carl Zeiss GmbH; Jena, Germany). Images were acquired at a lateral resolution of 333 nm. The integration time per spectrum was 0.1 s.

The spruce reference spectra were acquired with a 100 × air objective (Zeiss EC “Epiplan Neofluar” DIC, NA = 0.9, WD = 0.31 mm; Carl Zeiss GmbH, Jena, Germany). For each spectrum, 10 accumulations with an integration time of 0.5 s were used. Several spectra were taken to account for variations due to inhomogeneous drying.

For the larch samples, a very high auto-fluorescence was observed at 532 nm excitation, obstructing the Raman spectra. The references, as well as images, were consequently obtained at 785 nm excitation instead (near-IR, red, laser power 75 mW). The spectral ranges therefore differ between the two species, as the 785 nm spectra do not cover stretching vibrations involving hydrogen atoms (> 2700 cm^−1^). The spectrometer (UHTS400 spectrometer NIR) was also equipped with a back-illuminated and Peltier-cooled CCD camera (− 60 °C), with a 300 g/mm grating, resulting in a spectral resolution of 2.7 cm^−1^. Acquisition time was set to 1 s per spectrum to obtain a reasonable signal-to-noise ratio. Using the 100 × oil immersion objective, the maximal spatial (lateral) resolution of the larch images was 400 nm, but some samples where slightly oversampled with a pixel distance of 333 nm.

All images were acquired using a surface correction subroutine included in the WITec Control Suite 5 hardware control software (WITec GmbH, Lise-Meitner-Str. 6, D-89081 Ulm, Germany, http://www.WITec-instruments.com).

Reference spectra of larch extracts were acquired using the 100 × air objective. Each spectrum was acquired with 10 accumulations à 1 s integration time. The laser power for the water extracts was reduced to 30 mW to reduce burning of the sample. Because the average spectrum was still very noisy, it was smoothened with a Savitzgy-Golay filter (window size 6, order 2).

### Data analysis

Figure S1 in the SI shows the workflow of the data analysis described below. All data were processed and analyzed using MATLAB R2019a (The MathWorks, Inc., 1 Apple Hill Drive, Natick, MA 01,760–2098).

#### Preprocessing

Each image was inspected and image pixels with noticeable artifacts removed (only necessary for one larch image). Cosmic rays were removed using a third-order median filter along one spectral channel at a time.

Baseline correction of the images was done using an adaptation of the asymmetric least squares (ALS) algorithm^52^. Because of the heterogeneous backgrounds found in wood samples, the data were first clustered using k-means clustering. The parameters for the ALS were then adapted for each cluster. For spruce, λ = 1e5 in all clusters and *p* = 0.0001–0.0002. In larch, λ = 1e4-1e5 and *p* = 0.0001–0.0006.

To remove noise from the images, a principal component analysis (PCA) was done and the data reconstructed using 15 principal components (PCs) in spruce, and 10 PCs for the more noisy larch images. The first-derivatives of all spectra were calculated using a second-order Savitzky-Golay filter with window size 11. However, the zero-order data were used as-is after PCA reconstruction (no smoothening).

For the sake of data reduction, spectral ranges containing no peaks were removed, leaving the wavenumbers 250–1750 and 2530–3740 cm^−1^ for spectra obtained via 532 nm excitation, and 250–1750 cm^−1^ for spectra obtained at 785 nm excitation.

For the reference spectra, the same procedure was used, except for the denoising PCA. Then the data were normalized to equal length (2-norm of each spectrum).

#### Multiset image analysis

To obtain more consistent MCR-ALS solutions, the image data were grouped according to species and anatomical features (tracheids/rays/channels) and combined into multiset structures (column-wise augmentation). A summary of the model statistics for each multiset can be seen in Table S1.

Preliminary analysis showed that the models applied to the spruce data could be improved by preceding matrix normalization (each spectrum by its respective image norm, 2-norm). This step did not improve models with larch data, and was not used in this case.

#### Multivariate curve resolution—alternating least squares (MCR-ALS)

As schematically shown in Fig. S1a, the objective of image resolution is the decomposition of the raw image data (*D*) into concentration profiles (aka. distribution maps, *C*) and pure spectra (*S*^*T*^) of the constituents present in the imaged sample according to Eq. (1).

1 $$D = CS^{T} + E$$

MCR-ALS multiset analysis was performed on the multiset structures summarized in Table S1 following the MCR-ALS steps previously described^53^.

A first estimate of the number of components to look for was obtained via the eigenvalues of a PCA applied to each dataset (singular value decomposition, PLS Toolbox, Eigenvector Research, Inc. 196 Hyacinth Road, Manson, WA 98,831). The PC where the change in eigenvalues flattened out was seen as the minimum number of components. The MCR-ALS models were then built accordingly, but the final number of components was selected according to the appearance of pure spectra (i.e. least distorted after recovery from first derivatives) and clarity of distribution maps, along with the Lack of Fit (LOF) and variation explained (R^2^) of the MCR-ALS models (given in Table S1 of the SI). That is, we did not use any analytical method to calculate the number of components, but rather a combination of mathematical quality parameters and perceived biological meaningfulness. For all multisets, between 5 and 7 components were necessary for a good description of the variation space.

Then 5–7 purest zero-order spectra were selected with a 10% noise margin via the SIMPLISMA^36^ algorithm that is integrated in the MCR-ALS GUI 2.0. For the decomposition itself, preliminary analysis showed that using the first derivatives of our data resulted in clearer distribution maps, and better model statistics. Therefore, the derivatives of initial estimates were used together with the derivatized multiset to perform the iterative ALS optimization of matrices *C* and *S*^*T*^ under the following constraints: Non-negativity was applied only to the concentration profiles (distribution maps), but not to the spectra, because of the derivatives. Furthermore, the resolved component spectra in the **S**^**T**^ matrix were normalized using the Euclidean norm. Finally, the correspondence amongst species constraint was used for all larch datasets. It allows the user to introduce knowledge about presence or absence of certain components in one of the images of a multiset using a binary matrix. It is used when a component is present in some images of a multiset, but not in others of the same multiset. In this work, such components are marked with “absent” in the corresponding image (Fig. 5).

The drawback of using the derivatives was the interpretation of the chemical components, because the first derivatives are not as informative to the eye as the usual spectra and not all peak maxima have an inflection point at exactly zero. Therefore, we used the Moore–Penrose pseudoinverse^54^ of the concentration profiles obtained from MCR-ALS multiset analysis, multiplied with the normalized zero-order raw data to obtain reconstructed zero-order spectra, as shown in Eq. (2). The validity of this approach is detailed in the SI (Fig. S2).

2 $$S^{T} = \left( {C^{T} C} \right)^{ - 1} \cdot C^{T} D$$

### Principal component analysis of all MCR-ALS components and references

In order to get an overview of the complex relation of cell wall components and extractives obtained from MCR-ALS, a PCA was performed on the MCR-ALS components and reference spectra for each species separately (singular value decomposition, PLS Toolbox, Eigenvector Research, Inc. 196 Hyacinth Road, Manson, WA 98,831).

During preliminary analysis of the spruce data, the α-conidendrin and lumen water spectra were found to strain the model too much and were thus removed. In the case of larch, the lumen water, noise and CaF_2_ components were excluded from analysis. We also included several single spectra of oleoresin found in the lumen of various tracheids. For both PCA models, the first derivatives were used and cross-validation was done using venetian blinds with 10 splits and a blind thickness of 2.

### Data availability

The data generated and analyzed during the present study are available from the corresponding author on reasonable request.

## Supplementary Information

Supplementary Information.
