# Supplementary material for: Defensive strategies of Norway spruce and Kurile larch heartwood elucidated on the micron-level
Source: Sci Rep. 2021 Nov 15;11:22235. doi: 10.1038/s41598-021-01590-y (PMC8593066; doi:10.1038/s41598-021-01590-y)
Supplement: Supplementary file 1 — Supplementary Information. [file 41598_2021_1590_MOESM1_ESM.pdf]

Supplementary information to

Defensive strategies of Norway spruce and Kurile larch heartwood elucidated on the micron-level

Sophie Füchtner[1]*, Sara Piqueras[1,2], Lisbeth Garbrecht Thygesen[1]

[1] University of Copenhagen, Institute for Geoscience and Natural Resource Management, Rolighedsvej 23, 1955 Frederiksberg, Denmark

[2] MS-OMICS Bygstubben 9, DK-2950 Vedbæk, Denmark

*Corresponding author: sophie.fuchtner@ign.ku.dk

Section 1: Data analysis overview

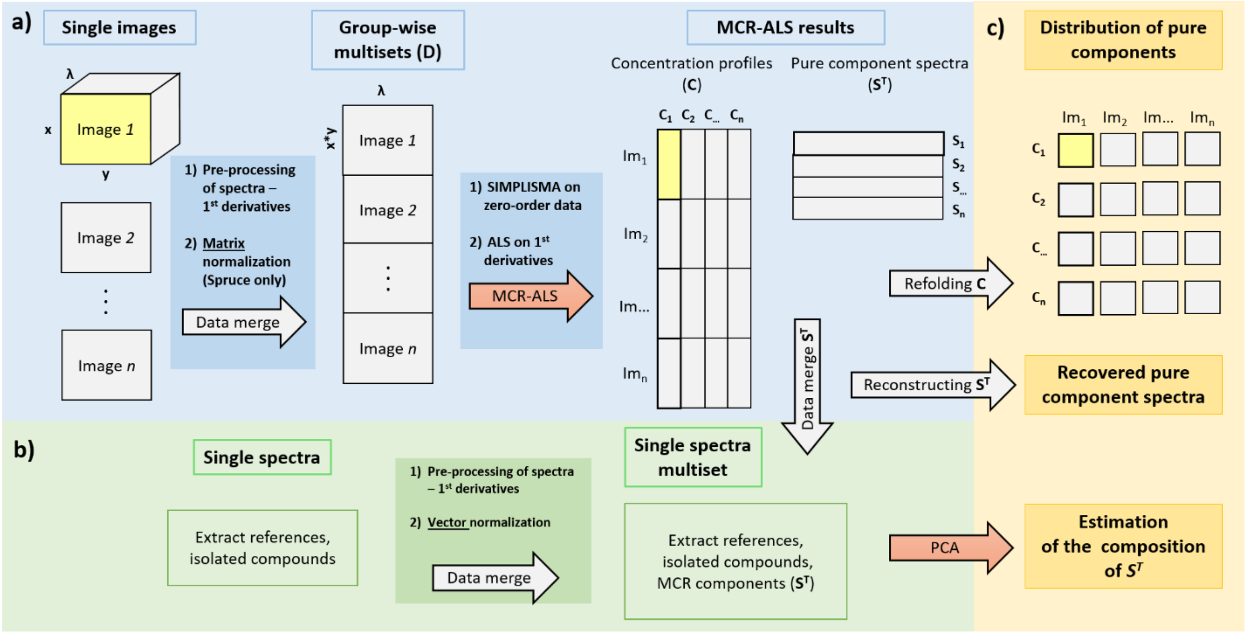

*Figure S1 - **Data structure and analysis workflow for a series of Confocal Raman images applied in this study**. **a)** Processing of the datasets obtained of Norway spruce and Kurile larch heartwood cells: After pre-processing, the data were merged group-wise into multisets (matrix D). Using MCR-ALS, they were decomposed into their pure component spectra (S^T) and the corresponding concentration profiles (C). **b)** Processing of reference data: Spectra of a series of extracts of the specimens, as well as pure reference compounds were obtained and pre-processed. These were analyzed jointly with the pure component spectra using PCA. **c)** Results obtained from the analysis: The distribution profiles of each of the components obtained from MCR-ALS, the corresponding recovered, zero-order component spectra and an estimate of the composition of native extractives and extracts.*

The workflow as described in the methods section of the main paper is schematically shown in **Figure S1**. The data analysis strategy we applied in this work was aimed at finding compounds within the Raman spectra that are present at extremely low concentrations compared to the matrix polymers (i.e., lignin, cellulose). This was achieved by a specially designed pre-processing chain, including a separate background correction for each image area and calculation of the first derivative of each spectrum, as detailed in the main paper. The SIMPLISMA algorithm was applied on the zero-order data in order to find a set of purest spectra in each dataset. The corresponding first derivatives were used as a starting point for the Multivariate Curve Resolution - Alternating Least Squares (MCR-ALS) algorithm (**Figure S1a**). The resulting concentration profiles were refolded into individual images, showing the distribution of the corresponding component, or presented in overlay modes. The pure components were obtained as first derivatives and the zero-order spectra were reconstructed using a pseudo-inverse matrix and the zero-order raw data (Equation (2) in the main text, **Figure S1c**). The validity of this approach is detailed in section 3. Furthermore, references obtained (**Figure S1b**) were analyzed with the pure component spectra using Principal Component Analysis (PCA) to clarify their composition (**Figure S1c**).

Section 2: Dataset details and model statistics

Table S1 shows a summary of the model details of each dataset used in the present study. The laser wavelength used is documented together with the number of images and image pixels of each dataset. Furthermore, pretreatment options, and number of components for each model are summarized. Finally, the model statistics are shown via the lack of fit (LOF) and the coefficient of determination (R^2).

Table S1 – Summary of the datasets analyzed by MCR-ALS, as well as model statistics. *For each dataset, following meta-data are shown: number (#) of images, total number of pixels, whether the set was image normalized, how many components were obtained by MCR-ALS, the lack of fit (LOF) and the coefficient of determination (R2). * The additional constraint correspondence amongst species was used for these multisets, as described in the methods.*

Dataset	excitation wavelength [nm]	# images	# pixels	image normalized	# MCR-ALS components	LOF [%]	R^2 [%]
Spruce 1 tracheids	532	3	29,619	yes	5	4.9	99.76
Spruce 2 rays	532	5	58,575	yes	7	5.2	99.73
Spruce 2 resin channels	532	3	52,164	yes	7	6.9	99.52
Larch tracheids	785	3	21,631	no	6*	5.74	99.67
Larch rays	785	5	40,251	no	6*	14.76	97.82
Larch resin channels	785	3	27,555	no	7*	8.6	99.27

Section 3: Validity of data reconstruction

Our preliminary data analysis showed that the use of derivatives improved the MCR-ALS results significantly. The drawback is that the spectra are not so easy to interpret, since the familiar band shapes are lost. Additionally, the x-axis is shortened by, in this case, 6 rel. cm^{-1} due to the Savitzky-Golay algorithm we used to compute the derivatives. Furthermore, the zero-crossings of the first derivatives do not coincide completely with peak maxima, which may also be due to the algorithm used and/or the smoothing. We therefore used the Moore-Penrose pseudoinverse[1] on the MCR-ALS results as a mathematical trick to obtain zero-order component spectra that can be interpreted easily.

Using a resin channel image of spruce as an example, we computed an average spectrum of 10 spectra selected from cell corners (lignin, **Figure S2a**), the secondary cell wall (cellulose, **Figure S2b**) and the center of a resin filled channel (oleoresin, **Figure S2c**). This was overlaid with the respective recovered zero-order components in **Figure S2**. As

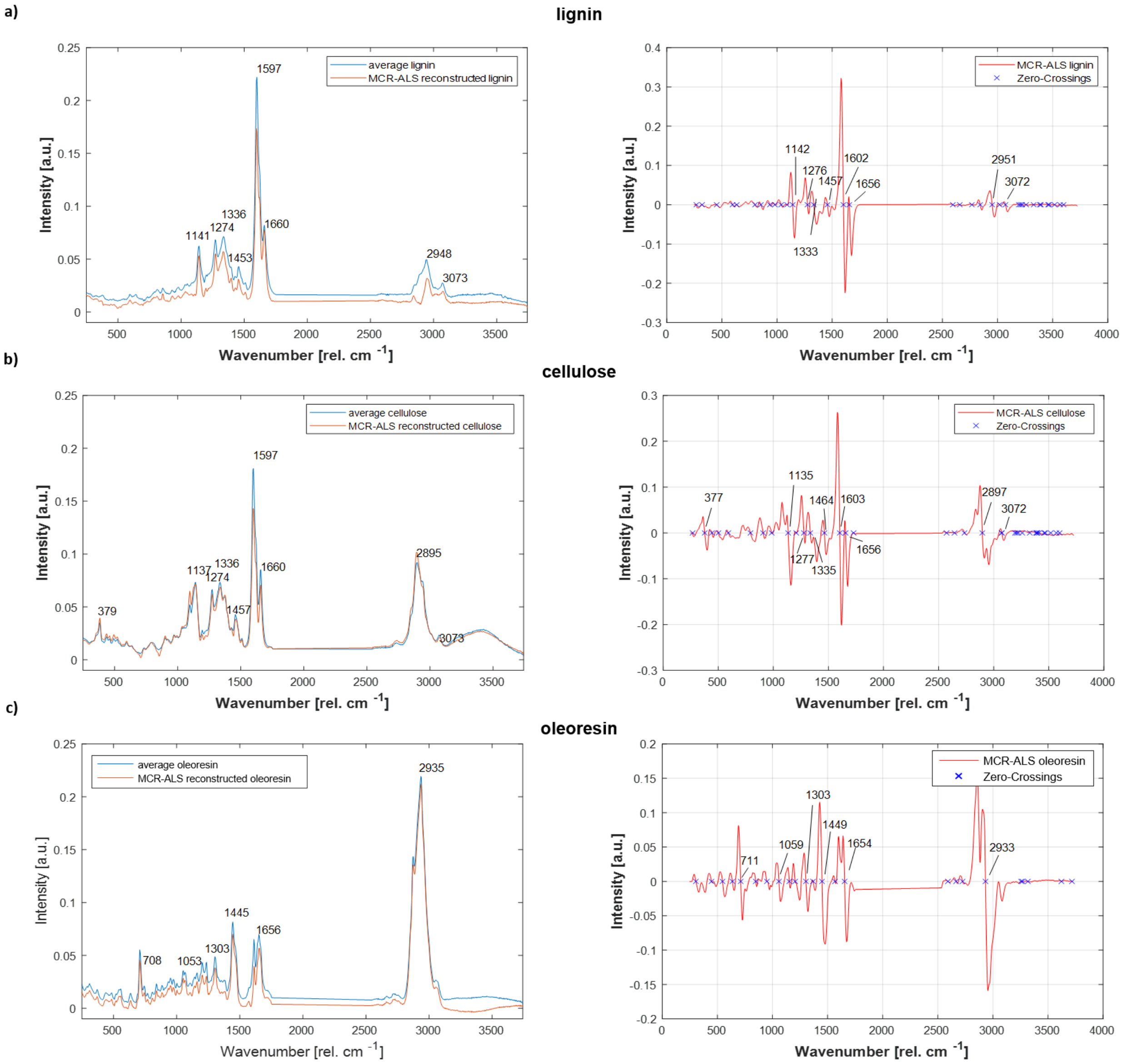

***Figure S2** - **Overlay of average spectra with the corresponding reconstructed zero-order MCR-ALS components** recovered using the Moore-Penrose pseudoinverse for **a)** lignin, **b)** cellulose and **c)** oleoresin. On the right-hand side, the original first-derivative components and band positions of selected zero-crossings are shown.*

can be seen, the spectra overlap perfectly well, which proves the validity of the approach. We show the respective components as first derivative, as obtained from MCR-ALS. Some band positions are shown to illustrate the issue of slightly deviating band positions.

Section 4: Cell wall components and background signals

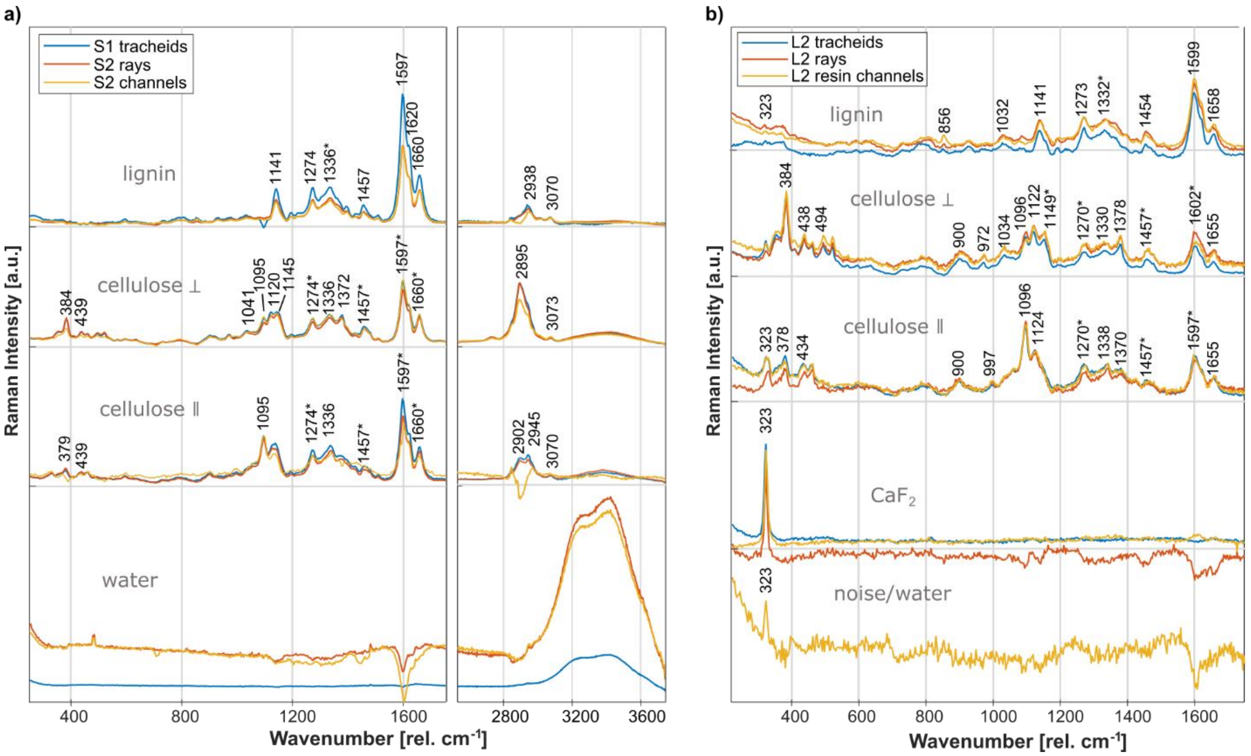

Figure S3 - MCR-ALS component spectra of the cell wall polymers and background-signals *identified in tracheid, ray and resin channel Raman images of Norway spruce and Kurile larch. Band positions labelled with " * " in the lignin spectrum correspond to cellulose bands and vice versa, as complete separation of these biopolymers into two separate model spectra was not possible.* ***a)*** *Spruce cell wall components (lignin, cellulose perpendicular and parallel to incident beam polarization), as well as the model spectrum representing the water introduced during sample preparation are shown.* ***b)*** *Larch cell wall components (as above), as well as CaF_2 signal from the cover glass and a residual noise/water spectrum.*

The CW polymers and various background signals are shown in **Figure S3** and the bands used for identification are summarized in **Table 1** of the main text. The spatial distribution maps of lignin and the two cellulose orientations were overlaid (**Figure S4**) and shown with a representative image of each dataset of both species. For the purpose of overlay, intensities were normalized to the range 0-1.

Lignin, typically most abundant in CCs and CML, was identified in all image sets based on the characteristic aromatic ring stretch at 1597/1599 cm^{-1} and the combination of C=C stretch of coniferyl alcohol and C=O stretch of coniferyl aldehyde at 1658/1660 cm^{-1} [2,3].

Because of the sensitivity of the Raman effect to crystal orientation, we obtained two components for cellulose. The major contribution came from crystalline cellulose microfibrils oriented perpendicular (⊥) to the incident beam polarization, known to constitute most of the thick S2 CW layer of tracheids. Typical bands are the carbohydrate C-H stretching band at 2895 cm^{-1} (spruce spectra) and the C-C-C ring breathing at 384

cm^{-1} [4]. The second, parallel (∥) cellulose component showed a marked decrease of the 384 cm^{-1} band, and an increase of the orientation sensitive C-C/C-O stretch at 1095/1096 cm^{-1} [5]. This component is known to be mostly represented in the outermost S1 CW layer. As discussed in the main text, lower amounts of parallel oriented cellulose was found in spruce resin channels. The inner CW of rays appears as a mix of cellulose (perpendicular) and lignin. Pits, allowing the transport of metabolites in between cells of various types, are apparent as dark spots in the ray cells (**Figure S4**). Note that residual lignin bands are present in both cellulose components and vice versa (marked by an asterix ("*") in **Figure S3**). This is caused by their high spatial correlation, which MCR-ALS cannot untangle.

Water from sample preparation was visible only in spectra recorded at 532 nm excitation (for spruce) with the large water O-H stretching band (>3000 cm^{-1} [6]). In the larch data, the CaF_2 cover-glass left a signal at 323 cm^{-1} [7], and also a noisy component, potentially water.

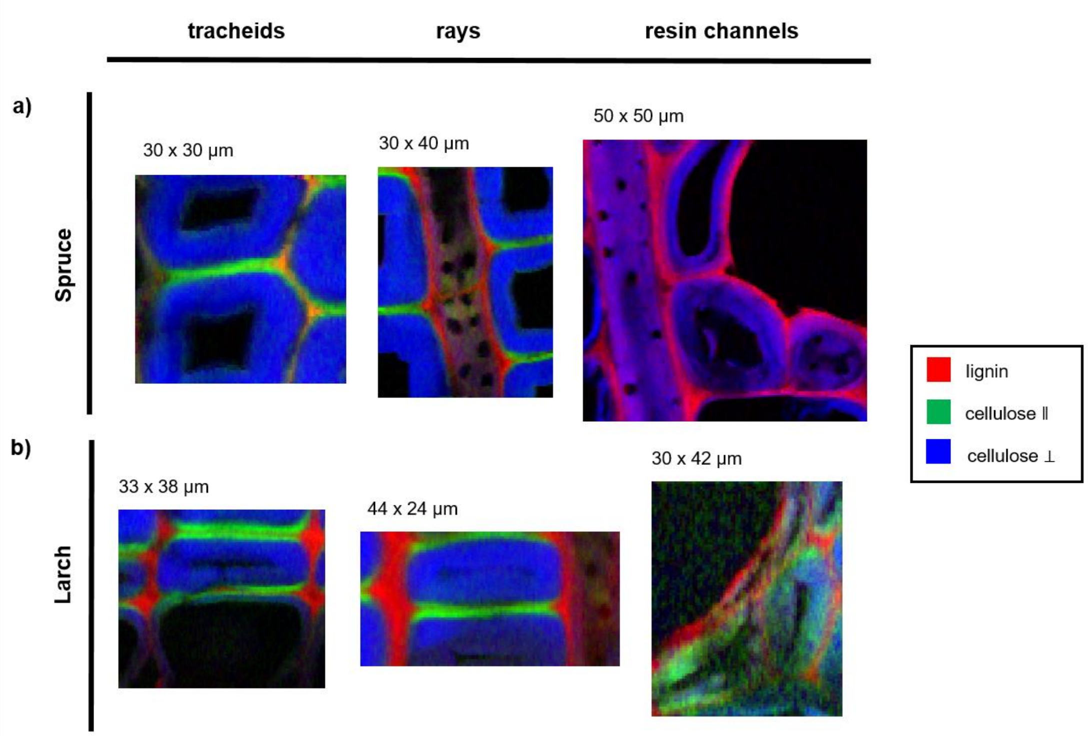

***Figure S4 - Overlay of the distribution maps of the cell wall components obtained from MCR-ALS analysis** of Raman images of cross-sections of spruce and larch tracheids, rays and resin channels. The main cell wall polymers are lignin (red), cellulose micro-fibrils with orientation parallel to the incident beam (green) and cellulose with perpendicular orientation to the incident beam (blue) **a)** Norway Spruce and **b)** Kurile larch. The data have been scaled to 0-1 for the purpose of this visualization.*

Section 5: Raman signature of extracted heartwood components

The spectra discussed in the following are shown in the main text for spruce and larch, respectively (**Figure 3a,b**). The solvents heptane, dichloromethane (DCM), ethanol and water were used sequentially. In a previous study, we assessed the free, monomeric extractive composition by GC-MS[8] and we used this information in the present study for the interpretation of the Raman spectra obtained. Additionally, reference spectra of the isolated lignans hydroxymatairesinol (HMR), lariciresinol and α-conidendrin were obtained. Due to the high structural similarity to lignin (monomers), an average cell corner (CC) spectrum was extracted from one of the spruce images. A spectrum was also recorded for commercial ArGal. Band positions are summarized in **Table 1** of the main text.

Both the GC-MS results presented earlier[8] and the Raman spectra presented here show that the hydrophobic heptane extracts were very similar between spruce and larch, although the RA composition differed. In both species, the presence of lipidic substances was evident from the high intensity C-H stretching bands. Vibrations specific for FAs were seen at 2852 (aliphatic C-H stretching) and 1303 and 1441-1465 cm^{-1} (various C-H bending modes)[9]. Unsaturated FAs may contribute to the shoulder at 1652 cm^{-1} (C=C stretch). The typically weak Raman signals of C=O and C-O stretches of FAs (~1730-1750 and ~860-900 cm^{-1}, respectively[9]) were not visible in these spectra. The presence of triglycerides was confirmed via their C-C stretching bands at 1063-1066 $cm^{-1}$and symmetric C-H_3 stretches at 2932 cm^{-1} [9], which could not be shown with the GC-MS method previously used[8], but is known from literature[10]. Additionally, the sharp maximum at 2905 cm^{-1} of the blue heptane spectrum (**Figure 3a**, main text) could be originating from a waxy aggregate in the sample that was not visible to the eye[11].

In the spruce heptane extracts, two intense bands at 1605 and 1636 cm^{-1} indicate the presence of compounds with aromatic or otherwise conjugated groups, which are enhanced at 532 nm excitation[2]. The only aromatic candidate known to be present in this sample in higher amounts is dehydroabietic acid, but its aromatic C=C stretch is expected at 1612 cm^{-1}. Abietic acid, a candidate with conjugated double bonds, is present at lower concentrations, and may thus contribute to the shoulder at 1652 cm^{-1}. Pimaric-type RAs have non-resonant (distant) C=C stretches, thus lower intensity, but could contribute to the 1636 cm^{-1} band (terminal C=C stretch), but the stretch of single C=C of the ring is not visible (1667 cm^{-1} [12]). A general skeletal vibration of RAs is seen at 712 cm^{-1}.

Since many RA-identifying bands are missing, another interpretation could be that the samples oxidized during the drying of the extract. Abietic-type RAs have been shown to form oxidation products with an intense doublet at 1601 and 1636 cm^{-1} (intermolecular hydrogen bonding[12]), and the presence of a keto-carbonyl vibration at 1714 cm^{-1}, which also visible in the spectrum.

In larch, the bands in the same region are much weaker (non-resonant at 785 nm) and indicate a mixture of pimaric-type RAs, abietic acid and small amounts of dehydroabietic

acid (**Figure 3b**, main text), reflecting our earlier GC-MS results[8]. Sterols and other diterpenoids were confirmed in the GC-MS study, but not identified in the Raman spectra of the extracts.

With increasing polarity of the extracting solvent, the aromatic character of the extract spectra increased, as seen by the increase of the C=C and C=O stretching region (>1590-1700 cm^{-1}), together with the decrease of aliphatic C-H bending and stretching vibrations of the hydrophobic lipids (~1444 cm^{-1} and >2800 cm^{-1}, respectively). The GC-MS study showed that while DCM extracts contained residues of the hydrophobic compounds, the ethanol extracts were almost purely composed of the more polar lignans in spruce and flavonoids in larch[8]. Again, this is confirmed by the Raman spectra presented here and in a principal component analysis (PCA) performed on extracts and extractive components (**Figure 6**), where the DCM extracts have the widest spread in the loadings, especially in spruce. The full PCA is presented in Section 6 below.

Based on the spectra of HMR, lariciresinol and α-conidendrin (lignans in **Figure 3a**), the presence of lignans in spruce is evident in the DCM and ethanol extracts, but also in the water extract, which could not be run on the GC-MS[8]. Note that the aromatic C=C stretching bands of the isolated lignans (1613-1620 cm^{-1}) appear narrower and at a slightly higher wavenumber compared to the extracts (1605-1609 cm^{-1}). Possible reasons may be the mixture of compounds present in the extracts and the micro-structure of the dried material. Despite the great structural similarity of lignans to lignin monomers, the lignin polymer (spruce CC lignin) has a more intense aromatic C=C stretch, located at 1597 cm^{-1}. In analogy to lignin, the most intense C-H stretching bands of lignans may be putatively assigned to OC-H_3 stretches and are located at ~2928-2935 cm^{-1} (except for α-conidendrin). In lignin, this band appears at 2942 cm^{-1}. Furthermore, the aromatic C-H stretching band of lignans at 3063-3067 cm^{-1} was reproduced in the extracts. This band appears at 3070 cm^{-1} for the lignin polymer.

The Raman spectra of the larch ethanol extract (**Figure 3b**), indicates a mixture of flavonoids by a broad combination band peaking at 1617cm^{-1} (C=O and aromatic C=C stretches), with a shoulder at 1640 cm^{-1}. Bands associated with the backbone include various aromatic ring modes and C-H bending vibrations at 1465, 1365 and 1295 cm^{-1} [32–34]. Bands associated with the OH-groups of taxifolin, the most abundant flavonoid in the sample, were found at 783 and 590 cm^{-1}. The backbone bands were also identified in the DCM extract, but not in the water extract. The latter only showed a weak contribution of C=O stretches from flavonoids (1615 cm^{-1}), and otherwise presented spectral features of isolated arabinogalactan. The latter were characterized by C-H_2 and C-H_3 bendings of carbohydrates at 1457, 1351 and 1262 cm^{-1}, C-C stretching vibrations at 1077 and ~1139 cm^{-1}., and glycosidic C-O-C stretches at 943 and 871 cm^{-1} [13].

Section 6: Overlay of extractive's distribution maps

An overlay of the extractive components found in Norway spruce (**Figure S5a,b**) and Kurile larch (**Figure S5c**) is shown below. The intensities have been normalized to 0-1 for the purpose of overlay.

Oleoresin, containing lignans in spruce, were identified in the lumen of rays, pits adjacent to rays and resin channels; lignan/lignin precursor signals were most intense in the middle lamellae on the lumen side of the resin channel. Aromatic waxes were found in the middle lamellae of tracheids, forming the torus of membranes within pits in rays, as well as lining the inner cell wall of epithelial cells. Additionally, a separate wax component was found in some pits of spruce rays.

In larch, oleoresin was found in the lumen of tracheids and in rays. In rays, it appears orange due to overlap with flavonoids. Flavonoids were found in the middle lamellae between all tracheid and parenchyma cells. Oleoresin, not containing flavonoids in larch,

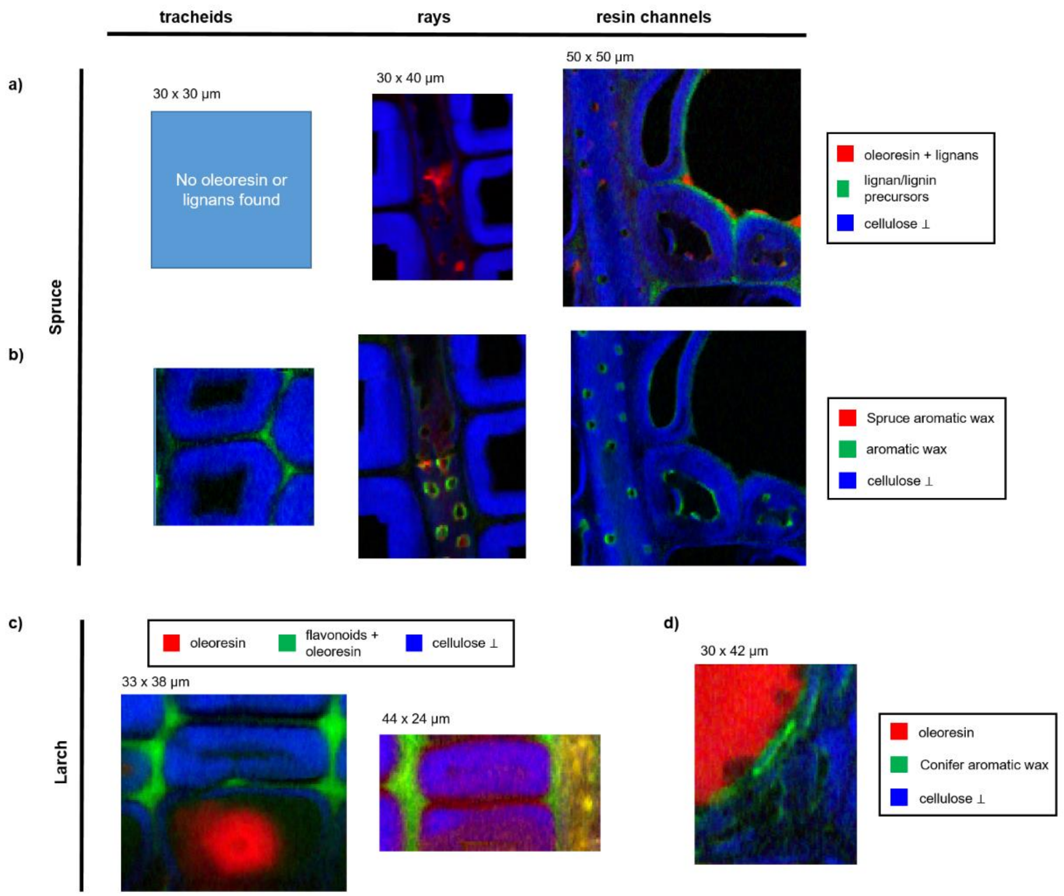

Figure S5 - ***Overlay of MCR-ALS distribution maps of the extractive components identified in Norway spruce and Kurile larch*** *shown together with the cellulose signal.* ***a)*** *Oleoresin and lignan/lignin precursor distribution of spruce;* ***b)*** *Two forms of aromatic wax in spruce have different distribution;* ***c)*** *Larch oleoresin and oleoresin mixed with flavonoids have distinct distributions* ***d)*** *Oleoresin and aromatic wax distribution in larch resin channels*

was also found in the lumen of resin channels. The aromatic wax component also lined the inner cell wall of larch epithelial cells.

Section 7: Correlation of references and component spectra assessed with Principal Component Analysis

To get a visual impression of the relationship between native extractives and the recorded references, we combined the MCR-ALS components of Norway spruce and Kurile larch heartwood images and the respective reference spectra into a dataset and analyzed it by PCA.

While individual bands seen in the references were found in the spectra that we isolated from the images via MCR-ALS, the overall shape of spectra often did not correspond completely. For a spectroscopic dataset, PCA calculates (often abstract) principal component spectra (loadings) that show how different variables co-vary among all the samples. Each consecutive principal component (PC) is orthogonal to the previous one and is calculated for the remaining sample variance. Each sample receives a score of how

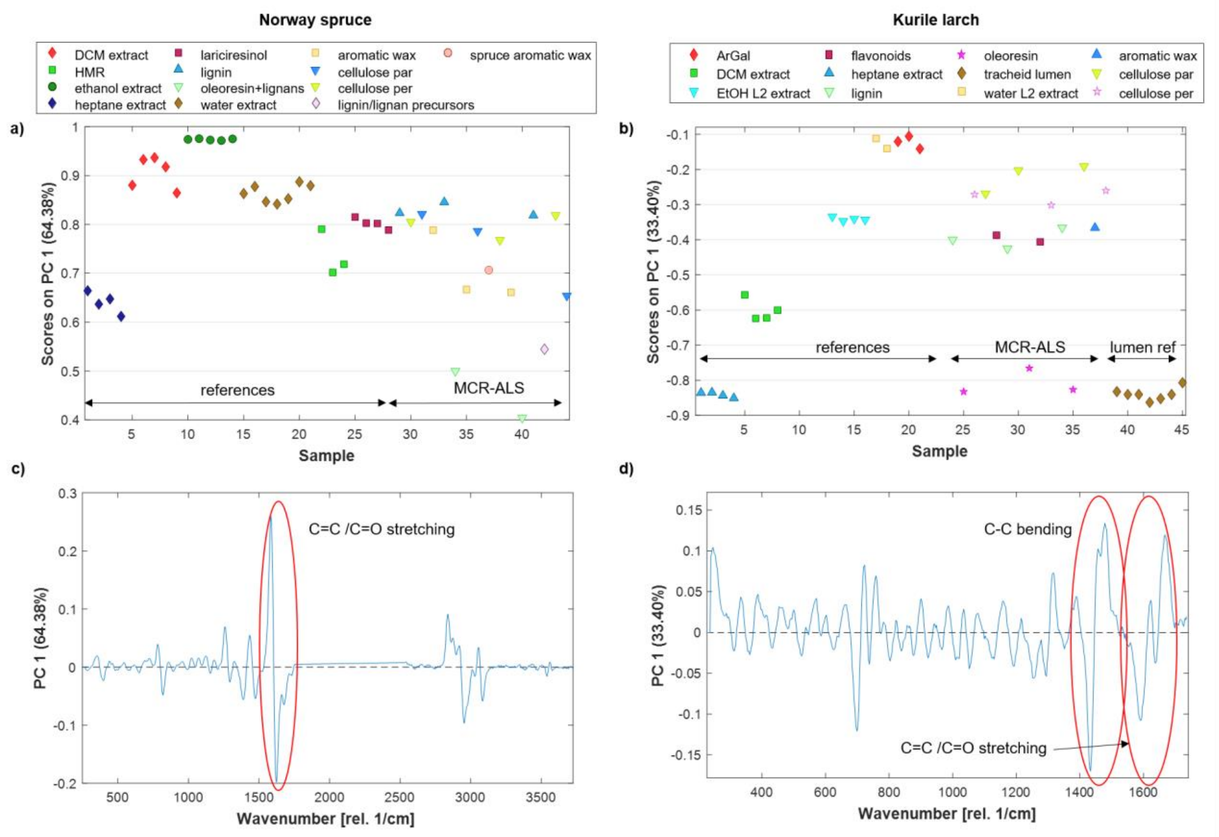

Figure S6 - Results of the first axis of the Principal Component Analysis *performed on the image components isolated by MCR-ALS and the reference extracts and substances acquired for this study.* ***a)*** *Sample scores and* ***c)*** *corresponding loadings for PCA of Norway spruce data.* ***b)*** *Sample scores and* ***d)*** *corresponding loadings for PCA of Kurile larch Raman data.*

well it is described by that PC. The sample scores of two principal components can be plotted against each other, thereby showing which samples are close to each other, and therefore have similar properties, i.e. peak positions and sizes.

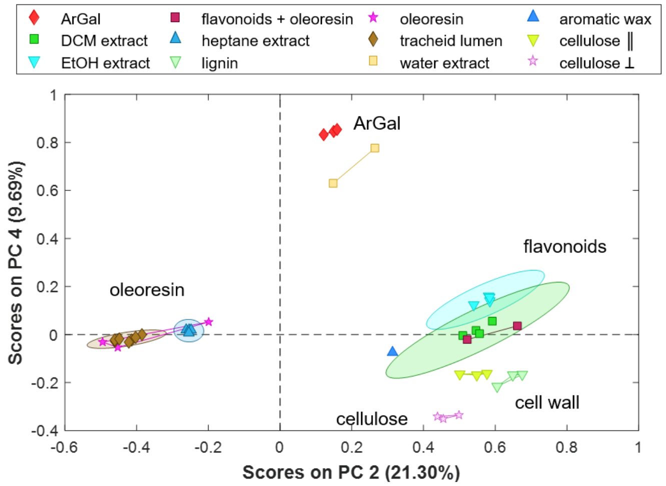

***Figure S7 –Scores plot of PC2 vs. PC4 of Kurile larch** additionally distinguishes arabinogalactan (ArGal) from cellulose.*

During preliminary analysis of the spruce data, the α-conidendrin and lumen water spectra were found to strain the model too much and were thus removed. In the case of larch, the lumen water, noise and CaF_2 components were excluded from analysis.

With three principal components (PCs) 82.77 % of the variation was explained with the spruce model (RMSEC 0.01541, RMSECV 0.02347), while 77.65 % were explained with four PCs for the larch dataset (RMSEC 0.0205, RMSECV 0.02587).

The major source of variation in both species is described by PC1. **Figure S6a,b** show the sample scores of PC1 for spruce and larch, respectively. The spectral features causing the sample placement can be seen via the variable loadings (**Figure S6c, d**).

For the spruce sample scores (**Figure S6a**), the ethanol extract (containing most concentrated lignans) scored highest, and the heptane extract and oleoresin component lowest. The variable loadings (**Figure S6c**) show that this is caused by the size of the aromatic bands (~1600 rel.cm^{-1}) and of the aliphatic C-H stretching area (2800-3100 rel.cm^{-1}). Among the larch samples (**Figure S6b**), the spectra and components with both, high amounts of hydrophobic compounds and aromatics, have the highest (negative) score, i.e. the dichloromethane (DCM) extract. Those lacking both score lowest (i.e. water extract and arabinogalactan). The corresponding bands can be viewed in **Figure S6d**.

The most informative loadings plot of PC2 vs. PC3 are presented in the main text (**Figure 6**). Due to space issues, only the most relevant information was mentioned there. For this reason, we would like to give a small overview at this point.

The ellipses shown in the **Figure 6** of the main text and **Figure S7** represent the probability area where samples with similar properties would be located on a 95 % confidence level (computed by PLS Toolbox).

For PC2 vs PC3 of both species, the heptane extracts and oleoresin components (hydrophobic) were placed on the opposite side of the axis compared to those with phenolic character (i.e. ethanol extracts). Because DCM extracts contained analytes of both regimes[8], they were located in between and showed the highest variability, i.e. largest confidence ellipses, especially in spruce.

The oleoresin components were closely associated with the heptane extracts in both species, confirming that the oleoresin component of spruce and larch consist of a mixture of fatty acids and esters and resin acids. For larch, single spectra of lumen content of several tracheids were also added to the PCA (**Figure 6** of the main text). Since these spectra associated with the heptane and oleoresin components, we confirm once again that some larch heartwood tracheids are filled with oleoresin. The presence of lignans in the oleoresin component of spruce is reflected by the slight shift towards the lignan references (**Figure 6a**).

For both species, all other extractive components were located in close vicinity to the cell wall polymers. This may hint at some connection to the cell wall. Interestingly, in the case of spruce (**Figure 6a**), the extractive components were located within the boundaries of the DCM confidence ellipse, while the cell wall polymers were not, supporting that these components are indeed extractives. Furthermore, the flavonoid component detected in larch (**Figure 6b**) associated with the DCM extract, indicating once again a mixture of flavonoids and hydrophobic oleoresin.

The lignan/lignin precursor component, found surrounding epithelial cells, was only weakly described by PC3, but was located in between the lignin and ethanol extract references. This supports the tentative assignment of this component as being precursor molecules of lignans and/or lignins.

The aromatic wax components were close to each other and the water extract in spruce, although the spectra look quite different. The composition of the spruce water extract was not clarified in our earlier study[8], but according to the spectrum (**Figure 3a** in the main text), lignans may be present. This was not confirmed with the component spectrum (**Figure 3c**). In larch (**Figure 6b**), the aromatic wax component was located in the same quadrant as the flavonoid-containing components and references, but was shifted towards the oleoresin components/references. Since no obvious signs for the presence of flavonoids was found in this component, this may be reflecting the mixture of aromatic compounds with aliphatic lipids.

Note that for both species, the water extract was located in the same quadrant as the cell wall polymers. It is probable that the extraction caused partial damage to the cell wall, and that cellulose and lignin monomers consequently were extracted[14]. This was confirmed with the GC-MS analysis performed on the larch water extract[8].

In **Figure S7**, the fourth PC of the larch dataset is shown. It resulted in the distinction of cellulose from arabinogalactan. The water extracts grouped with the arabinogalactan, confirming the presence of arabinogalactan in this extract.

References

1. Barata JCA, Hussein MS. The Moore–Penrose Pseudoinverse: A Tutorial Review of the Theory. *Brazilian J Phys*. 2012;42(1-2):146-165. doi:10.1007/s13538-011-0052-z

2. Bock P, Gierlinger N. Infrared and Raman spectra of lignin substructures: Coniferyl alcohol, abietin, and coniferyl aldehyde. *J Raman Spectrosc*. 2019;50(6):jrs.5588. doi:10.1002/jrs.5588

3. Gierlinger N. Revealing changes in molecular composition of plant cell walls on the micron-level by Raman mapping and vertex component analysis (VCA). *Front Plant Sci*. 2014;5(JUN):306. doi:10.3389/fpls.2014.00306

4. Edwards HGM, Farwell DW, Williams AC. FT-Raman spectrum of cotton: a polymeric biomolecular analysis. *Spectrochim Acta Part A Mol Spectrosc*. 1994;50(4):807-811. doi:10.1016/0584-8539(94)80016-2

5. Gierlinger N, Schwanninger M. Chemical imaging of poplar wood cell walls by confocal Raman microscopy. *Plant Physiol*. 2006;140(4):1246-1254. doi:10.1104/pp.105.066993

6. Baschenko SM. On Raman spectra of water, its structure and dependence on temperature. *Semicond Phys Quantum Electron Optoelectron*. 2011;14(1):77-79. doi:10.15407/spqeo14.01.077

7. Gee AR, O'Shea DC, Cummins HZ. Raman scattering and fluorescence in calcium fluoride. *Solid State Commun*. 1966;4(1):43-46. doi:10.1016/0038-1098(66)90102-5

8. Füchtner S, Brock-Nannestad T, Smeds A, Fredriksson M, Pilgård A, Thygesen LG. Hydrophobic and Hydrophilic Extractives in Norway Spruce and Kurile Larch and Their Role in Brown-Rot Degradation. *Front Plant Sci*. 2020;11(June). doi:10.3389/fpls.2020.00855

9. Czamara K, Majzner K, Pacia MZ, Kochan K, Kaczor A, Baranska M. Raman spectroscopy of lipids: A review. *J Raman Spectrosc*. 2015;46(1):4-20. doi:10.1002/jrs.4607

10. Nisula L. Wood Extractives in Conifers A Study of Stemwood and Knots of Industrially Important Species. 2018.

11. Sasani N, Bock P, Felhofer M, Gierlinger N. Raman imaging reveals in-situ microchemistry of cuticle and epidermis of spruce needles. *Plant Methods*. 2021;17(1):1-15. doi:10.1186/s13007-021-00717-6

12. Beltran V, Salvadó N, Butí S, Cinque G, Pradell T. Markers, Reactions, and Interactions during the Aging of Pinus Resin Assessed by Raman Spectroscopy. *J Nat Prod*. 2017;80(4):854-863. doi:10.1021/acs.jnatprod.6b00692

13. De A, Malpani D, Das B, Mitra D, Samanta A. Characterization of an arabinogalactan isolated from gum exudate of Odina wodier Roxb.: Rheology, AFM, Raman and CD spectroscopy. *Carbohydr Polym*. 2020;250(August):116950. doi:10.1016/j.carbpol.2020.116950

14. Örså F, Holmbom BR. A Convenient Method for the Determination of Wood Extractives in Papermaking Process Waters and Effluents. *J Pulp Pap Sci*. 1994;20(12):361-366.
